# miRConnect 2.0: identification of oncogenic, antagonistic miRNA families in three human cancers

**DOI:** 10.1186/1471-2164-14-179

**Published:** 2013-03-15

**Authors:** Youjia Hua, Niels Larsen, Shanker Kalyana-Sundaram, Jørgen Kjems, Arul M Chinnaiyan, Marcus E Peter

**Affiliations:** 1 Feinberg School of Medicine, Division Hematology/Oncology, Northwestern University, Chicago, 60611, USA; 2 Department of Molecular Biology, Aarhus University, Århus, Denmark; 3 Michigan Center for Translational Pathology, Ann Arbor, MI, 48109, USA

**Keywords:** Oncogenes, Tumor suppressors, Gene array, microRNA (miRNA) groups, NCI60 cell lines

## Abstract

**Background:**

Based on their function in cancer micro(mi)RNAs are often grouped as either tumor suppressors or oncogenes. However, miRNAs regulate multiple tumor relevant signaling pathways raising the question whether two oncogenic miRNAs could be functional antagonists by promoting different steps in tumor progression. We recently developed a method to connect miRNAs to biological function by comparing miRNA and gene array expression data from the NCI60 cell lines without using miRNA target predictions (miRConnect).

**Results:**

We have now extended this analysis to three primary human cancers (ovarian cancer, glioblastoma multiforme, and kidney renal clear cell carcinoma) available at the Cancer Genome Atlas (TCGA), and have correlated the expression of the clustered miRNAs with 158 oncogenic signatures (miRConnect 2.0). We have identified functionally antagonistic groups of miRNAs. One group (the agonists), which contains many of the members of the miR-17 family, correlated with c-Myc induced genes and E2F gene signatures. A group that was directly antagonistic to the agonists in all three primary cancers contains miR-221 and miR-222. Since both miR-17 ~ 92 and miR-221/222 are considered to be oncogenic this points to a functional antagonism of different oncogenic miRNAs. Analysis of patient data revealed that in certain patients agonistic miRNAs predominated, whereas in other patients antagonists predominated. In glioblastoma a high ratio of miR-17 to miR-221/222 was predictive of better overall survival suggesting that high miR-221/222 expression is more adverse for patients than high miR-17 expression.

**Conclusion:**

miRConnect 2.0 is useful for identifying activities of miRNAs that are relevant to primary cancers. The new correlation data on miRNAs and mRNAs deregulated in three primary cancers are available at miRConnect.org

## Background

miRNAs are small noncoding RNAs that regulate gene expression by causing degradation of mRNAs or by inhibiting protein translation [1]. The emerging conventional view is that miRNAs are deregulated in all human cancers [2]. miRNAs act by targeting a short sequence (the seed match) in the 3'UTR of targeted mRNAs. Numerous algorithms have been developed that allow prediction of miRNA targets. However, the prediction accuracy is low and includes a large number of false positives and false negatives [3]. From our analysis of the miR-200 family of miRNAs and its biological activities we realized that the combination of differentially expressed genes (both up and downregulated genes) can be used to deduce the biological activities of miRNAs [4]. We and others found that miR-200 regulates the epithelial-to-mesenchymal transition (EMT) by suppressing the expression of mesenchymal genes and inducing expression of epithelial genes [5-8]. We recently developed a new method (summed (s)PCC) to better correlate miRNAs and gene expression with the goal of predicting biological activities of miRNAs. We tested this method by analyzing gene array and miRNA expression data sets available for the 60 cell lines of the drug screen panel at the National Cancer Institute (NCI60 cells) [4]. By comparing genes that positively correlate with miRNAs and miRNA families we clustered miRNAs into functional groups. One group of miRNAs, which was preferentially expressed in epithelial cells, contained all 5 members of the miR-200 family. Another group antagonized the members of the epithelial group of miRNAs. In addition, we identified and validated three other miRNAs that regulated EMT: miR-7, miR-203 and miR-375 [4]. The data sets are available in a searchable form at miRConnect.org.

Multiple studies have reported correlations of miRNA and mRNA data in NCI60 cell lines [9-14] as well as in primary tumors [15-29]. Most of these studies identified targets of individual miRNAs in a specific cancer background, while some also identified miRNA functions across multiple tumor origins [25-29]. However, the primary goal of most studies was to predict targets of individual miRNAs and to use this information to predict function. In contrast, our approach is independent of miRNA target predictions.

Certain miRNAs (oncomiRs) can act as tumor suppressors or as oncogenes [30]. Not all oncomiRs are deregulated in all cancers suggesting that miRNAs have specific activities in different cancers and/or cancer stages. As well, different tumorigenic activities found in cancer could even be antagonistic. A putative example of such antagonistic activities might be cell proliferation and "stemness". Thus, we predicted the existence of functionally antagonistic, oncogenic miRNAs. To test this hypothesis in a cancer relevant context, we extended our analysis using the sPCC method to primary cancer data sets available at The Cancer Genome Atlas (TCGA): ovarian cancer (OvCa), glioblastoma multiforme (GBM), and kidney renal clear cell carcinoma (KIRC). The new data have now been incorporated into version 2 of miRConnect. Using the sPCC analysis and by comparing the expression of miRNA and mRNAs with expression data for 158 well described oncogenic signatures, we have identified large groups of miRNAs that antagonize each other in cancer cells. Two of these antagonizing miRNA groups are considered to be oncogenic. One group (the "agonists") is dominated by members of the miR-17 gene clusters, the other (the "antagonists") contains miR-221 and miR-222. Pathway analysis suggests that both agonists and antagonists are tumorigenic and regulate different cancer relevant signaling pathways. In GBM, we found that patients in whom the expression of the antagonists predominates have poorer overall survival, which suggests that while both miR-17 and its relatives and miR-221/222 may be good biomarkers for detecting tumor cells, high miR-221/222 expression maybe a better predictor of poor outcome.

## Results

### Identification of antagonistic miRNAs in the NCI60 cells

**Figure 1:**
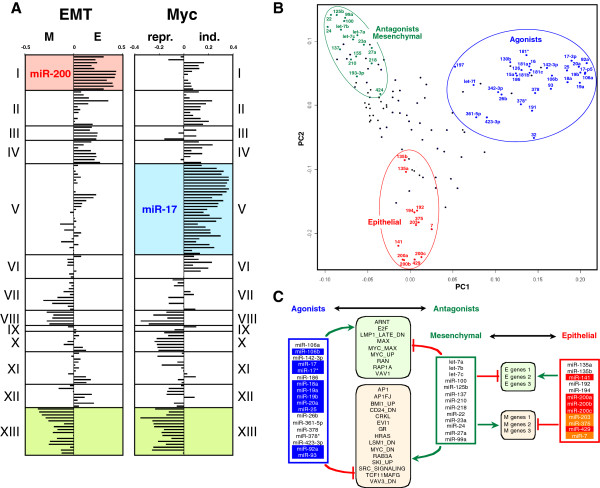
**Identification of antagonistic miRNA clusters using the NCI60 data sets.** (**A**) Correlation of gene signatures with 136 miRNAs grouped into 13 miRNAs clusters according to their positively correlating genes as recently described [4]. Left panel, average EMT gene signature (see Methods). M, mesenchymal genes; E, epithelial genes. Right panel, c-Myc-repressed (repr.) and c-Myc-induced (ind.) genes. Three clusters that had highly significant correlations in the Wilcoxon rank analysis (p < 10^-7^) are highlighted in different colors. The X-axis displays a factor that indicates the level of correlation with the gene sets as explained in the Method section. (**B**) PCA (Principal Component Analysis) of the 136 miRNAs in A. The miRNAs identified in the three clusters are circled in similar colors as in A. (**C**) Correlation network based on miRNA groups and gene signatures (E genes, M genes, and oncogenic signatures). Only correlations that were found in the analysis of positively and negatively correlating genes are shown (see Additional file 4: Table S4 and Additional file 6: Table S5). Red box, epithelial; blue box, agonistic; green box, mesenchymal and antagonistic. miRNAs: red, miR-200 family; blue, miR-17 family; orange, other EMT-related miRNAs recently identified [4] (miR-7, miR-203, and miR-375). EMT genes: light green box, E genes; light red box, M genes. Oncogenic signatures: light green, positively correlated with agonistic miRNAs; light red, negatively correlated with agonistic miRNAs. Epithelial specific miRNAs are highlighted in red and members of the miR-17 family of miRNAs are highlighted in blue.

We recently used miRNA and mRNA data sets available for the NCI60 cells to identify groups of miRNAs with similar biological functions [4]. For this purpose, we have developed a novel PCC analysis (summed (s)PCC) that mimics an in silico titration assay. Using this method, we found that 136 miRNAs significantly expressed in at least 30 of the 59 NCI60 cell lines clustered not only according to their seed sequences, genomic organization, and tissue specific expression but also according to their biological function. When the miRNAs were clustered according to the genes with which they were positively correlated, a total of 13 clusters were defined (Figure 1A) using a threshold of 12.5% correlating genes to define a cluster as described [4]. miRNAs in cluster I contained the 5 miRNAs of the miR-200 family known to be strong EMT regulators. These data were based on three independent EMT signatures: EMT signature 1, normal tissue induced to undergo EMT by addition of TGFβ, EMT signature 2, RAS transformed cells induced to undergo EMT by addition of TGFβ, and EMT signature 3, metaplastic versus ductal breast cancer. Since the three signatures were very similar, we based the analysis on the average of the three signatures (Figure 1A). Again, cluster I was correlated with the epithelial genes in the combined EMT signature (p < 10^-7^). In addition to the miR-200 family members, the EMT signature also contained miR-7, miR-203, and miR-375, which we previously identified and validated as novel EMT regulating miRNAs [4]. With the same level of significance, we now identified miRNAs in cluster XIII as miRNAs that correlated with the expression of mesenchymal genes. These data suggest that the miRNAs in cluster XIII in the NCI60 cell lines may have opposing functions when compared the miRNAs in cluster I. In addition, we previously identified the miRNAs in cluster V as miRNAs that positively correlated with c-Myc induced genes and negatively correlated with c-Myc repressed genes [4]. This was also evident when we plotted the data using a combined factor of c-Myc regulation (Figure 1A, see method section for details). We also showed recently that miRNAs in cluster V are most strongly regulated by c-Myc. The most prominent miRNA family present in cluster V are family members of the miR-17 ~ 92 cluster and its related paralogs, the miR-106 ~ 363 and miR-106 ~ 25 clusters. All of these clusters of miRNAs are known to be regulated by c-Myc [24,31,32]. Interestingly, the cluster of miRNAs most negatively correlating with c-Myc induced genes and strongly correlating with c-Myc repressed genes are in cluster XIII, which contains the miRNAs that correlated with mesenchymal genes. miR-17 and all its homologues are widely recognized as highly oncogenic miRNAs [33], while the cluster I epithelial specific miRNAs are viewed as tumor suppressive in most solid cancers [34].

So how is it possible that the cluster XIII miRNAs can be antagonists to the epithelial miRNAs in cluster I and at the same time antagonists to the oncogenic miRNAs in cluster V? This result suggested that in the context of cancer miRNAs cannot be simply divided into oncogenic and tumor suppressive miRNAs. This insight became more evident when we reassessed a principal component (PC) analysis of miRNAs based on the genes with which they positively correlated [4] (Figure 1B). In this analysis, miRNAs grouped in a two dimensional space according to the similarity with which they correlated with the 18,000 genes available for the NCI60 cells. Of the 136 PCs, the combination of the first two described about 50% of all variance among miRNAs (data not shown). All miRNAs in the three clusters (I, V and XIII) in Figure 1B are labeled in the colors shown in Figure 1A. The PCA clustering produced the shape of a three bladed propeller in which the members of the three clusters occupied the tips of the three blades. The first PC separated agonists from mesenchymal antagonists, while the second PC separated epithelial miRNAs from both agonists and mesenchymal antagonists. The three fold symmetry of the PCA plot suggested that the miRNA world is divided in at least three cancer relevant activities whereby epithelial miRNAs are antagonized by mesenchymal miRNAs, which are c-Myc repressed, and epithelial miRNAs functionally antagonize c-Myc induced miRNAs, most notably members of the miR-17 family. In order to assess the nature of the correlating cancer specific genes for each miRNA cluster, we selected 158 different oncogenic gene signatures each containing from five to several hundred different genes (Additional file 1: Table S1). In order to increase the stringency, we then selected the set of overlapping genes and miRNAs that either positively (Additional file 2: Table S2) or negatively (Additional file 3: Table S3) correlated with each other. We found that genes in 9 oncogenic signatures positively correlated and 15 signatures negatively correlated with a group of agonistic miRNAs that were dominated by members of the miR-17 family (p < 0.001) (Figure 1C). A number of miRNAs belonging to the "mesenchymal" cluster showed a correlation that was exactly opposite to these agonistic miRNAs. We called these antagonists. The antagonist miRNA cluster (which contained three members of the let-7 family) negatively correlated with epithelial genes (in all three EMT signatures) and positively correlated with mesenchymal genes. Exact functional opposites were found in the miRNA group that contained the miR-200 families plus miR-7, miR-203, and miR-375 (Figure 1C). In summary, the data suggest that in cancer cell lines miRNAs can be grouped according to their function, and at least three major, mutually antagonistic functions can be assigned.

### The primary cancer data sets

While the data obtained from NCI60 cells identified miRNA groups that could function as antagonists, the analysis had a number of limitations: 1) Because the analysis was based only on cancer cell lines without comparison to normal tissue, it was uncertain how relevant these connections were to cancer. 2) The data were based on a high quality but limited set of only 208 miRNA quantified by real time PCR. 3) The relevance of the findings to primary human cancer remained unclear. To address all three shortcomings, we turned to the large database of The Cancer Genome Atlas (TCGA). At the time of our analysis, the TCGA database contained information on 19 solid cancers. Additional file 4: Table S4, column 1 lists the number of available tumor samples for each cancer. Column 2 shows the number of samples for which both mRNA and miRNA data were available. In order to limit our analysis to high quality data, we elected not to consider any patient sample with a tumor content of less than 70%. Tumor content in the TCGA samples is determined by a pathologist who evaluates a slice from the top and a slice from the bottom of the tissue block used for RNA isolation to quantify the percent tumor content. In Additional file 4: Table S4, column 3, the number of samples is listed for which information from the bottom and top of the block was available. Column 4 gives the number of patient samples with more than 70% tumor content (average of top and bottom analysis >70%). To maintain a robust sample size, we did not further consider cancers with fewer than 100 patient samples. Only 4 cancers remained: breast cancer (BRCA), glioblastoma (GBM), ovarian cancer (OvCa), and clear cell renal cancer (KIRC). For each of the cancers, pathology data were available (i.e., tumor stage, grade or histology). In order to have a homogenous patient population and to give the analysis sufficient statistical power, we focused on the largest group of patients with similar features for each cancer. In breast cancer, we selected the 143 infiltrating ductal ER positive BRCA. For GBM, we included 353 patients with untreated primary GBM. In KIRC we selected all 142 treated patients with tumor grade G2-G4 and all tumor stages. In OvCa we selected 320 untreated patients with primary cancer in the ovaries, grade 3 and stages IIIB, IIIC, and IV. We proceeded with the analysis including these four solid cancers. However, early analysis indicated that the breast cancer data sets were not giving consistent correlations. This was likely because the breast cancer samples corresponded to more than one disease [35]. Therefore, we elected to perform the analysis on three cancers: OvCa, GBM, and KIRC.

### miRConnect 2.0: identification of antagonistic miRNAs in primary cancers

The number of genes and miRNAs for each cancer with expression data provided in the TCGA data sets is given in Additional file 4: Table S4. In Additional file 4: Table S4 column 7 the number of normal controls is given. In order to focus only on genes and miRNAs that are cancer relevant we excluded for each cancer all genes and miRNAs which were less than 1.5 fold deregulated when their average expression in tumor and normal samples were compared. The number of miRNAs/mRNAs deregulated for each cancer was 173/2046 (for OvCa), 121/3890 (for GBM) and 260/9288 (for KIRC). The number of genes and miRNAs that were similarly deregulated in all three cancers was low (Additional file 5: Figure S1) suggesting major cancer-specific differences. The data and correlation analysis between all miRNAs and mRNAs deregulated >1.5 fold in the three cancers can be accessed in a searchable form at miRConnect.org or miRConnect.net. In addition to the data on NCI60 cells based on a set of real-time PCR miRNA data (miRConnect-Q) and LNA array data (miRConnect-L), data on ovarian cancer are found under miRConnect-OvCa; GBM is under miRConnect-GBM, and renal cancer is found under miRConnect-KIRC.

**Figure 2:**
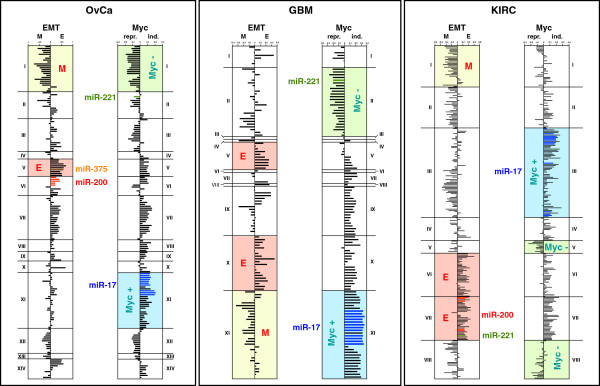
**Functional clusters of deregulated miRNAs and associated gene signatures in three primary cancers.** Left panels, EMT regulated genes; Right panels, c-Myc regulated genes. Clusters with highly significant positive correlations (p < 10^-4^ for OvCa and GBM; p < 10^-5^ for KIRC) are highlighted in different colors. Location of selected miRNAs are shown. For a complete order of miRNAs for all three cancers clustered according to either positively or negatively correlating genes see Additional file 6: Table S5.

To test whether the genes and miRNAs specifically deregulated in each cancer were functionally connected, we correlated the expression of the deregulated miRNAs with expression of the mRNAs using the sPCC method previously developed to analyze the NCI60 data. When the cluster analysis of the deregulated miRNAs was performed with the positively correlating cancer-specific mRNAs using the same 12.5% threshold to identify clusters (see [4] and Figure 1A), the 173 miRNAs in OvCa fell into 14 clusters; for GBM we found 11 clusters, and for KIRC 8 clusters (Additional file 5: Figure S2). The same analysis was repeated with all genes that negatively correlated with the expression of the miRNAs. The complete lists of miRNAs in each of the identified clusters in all analyses (NCI60 plus three cancers) are shown in Additional file 6: Table S5. The genes that correlated with each cluster of miRNAs were compared to the three EMT signatures and the signature of c-Myc regulated genes, and a number of miRNA clusters were found to correlate with EMT and c-myc regulated genes (Figure 2).

In contrast to the NCI60 cell analysis in which all members of the miR-200 family correlated with epithelial (E) genes (highlighted in red in Figure 1A), the situation in the primary cancers was more complex. In OvCa, the cluster most significantly correlating with the E genes was cluster V. While this cluster contained the epithelial miRNA miR-375, all 5 members of the miR-200 family were part of cluster VI, which did not correlate with the E genes as significantly as cluster V miRNAs. In GBM, none of the miR-200 family members are deregulated in cancer and were not part of the analysis. Two clusters (V and X) significantly correlated with the expression of E genes suggesting that in GBM miRNAs other than miR-200, miR-7, miR-203, or miR-375 regulate the epithelial nature of the cancer cells. Finally, in KIRC all miR-200 family members were found to be part of cluster VII, which together with cluster VI miRNAs most significantly correlated with E genes. For each of the three cancers, one cluster was found to contain miRNAs that significantly correlated with the expression of mesenchymal (M) genes (highlighted in yellow in Figure 2). For OvCa this was cluster I; for GBM it was cluster XI, and for KIRC it was cluster I. However, there was no overlap among these miRNAs between any of the three cancers (data not shown), again suggesting that this activity was regulated by different cancer-specific miRNAs.

**Figure 3:**
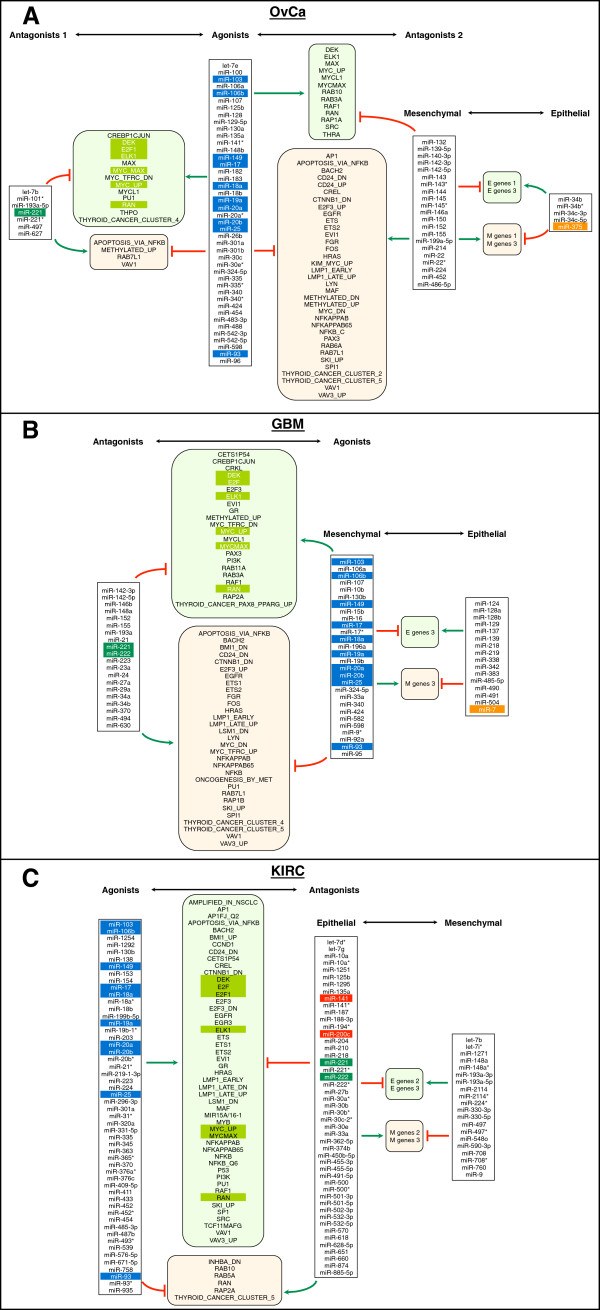
**A correlation network of miRNA groups and gene signatures in three primary cancers.** (**A**) OvCa. (**B**) GBM. (**C**) KIRC. miRNAs: blue, miR-17 family; dark green, miR-221/222 family; red, EMT-related miRNAs (miR-200 family); orange, other EMT-related miRNAs (miR-7, 203 or 375). Oncogenic and EMT signatures (in rectangular boxes with round corners): light green, positively correlating with agonistic miRNAs; light red, negatively correlating with agonistic miRNAs; oncogenic signatures highlighted with light green, overlapping signatures among three primary cancers.

The situation was very different for c-Myc induced genes. In all three cancers, the cluster that most significantly correlated with the expression of c-Myc induced genes contained a large number of miR-17 miRNAs: cluster XI in OvCa, cluster XI in GBM, and cluster III in KIRC (highlighted in blue in Figure 2). These significantly overlapped with the agonistic miRNAs previously identified in the NCI60 cell analysis (Figure 1). This raised the question of whether more than two antagonistic miRNA cluster occurred in primary cancer cells, as had been observed in NCI60 cells. Therefore, we performed an integrated miRNA/mRNA analysis to determine if any of the different miRNA clusters identified in the three cancers correlated with gene expression profiles associated with the three EMT signatures or the 158 known oncogenic gene signatures (Additional file 7: Table S6 and Additional file 8: Table S7). To increase the stringency, we performed two analyses for each comparison, one in which the clustering was based on positively correlating genes and one based on negatively correlating genes. The goal of this analysis was to identify miRNA clusters that were positively correlated with a number of gene signatures (the agonists) and other clusters that would have an opposite correlation (the antagonists) with the same gene signatures. Details on the analysis are found in Methods and in Additional file 8: Table S7. The results of the complete analysis for each cancer are summarized in Figure 3A-C. All significantly antagonizing miRNA clusters are shown. In all three cancers, the major agonistic miRNA group contained members of the miR-17 clusters (highlighted in dark blue). Interestingly, in GBM and KIRC this agonistic group antagonized an epithelial miRNA group, but in OvCa it antagonized a mesenchymal group of miRNAs.

The agonistic group of miRNAs shares 8 members of the miR-17 cluster plus miR-103 and miR-149 in all three cancers, based on the analysis of both positively and negatively correlating genes (Additional file 9: Table S8-1). Interestingly, the 8 miR-17 family members are found in all three miR-17 gene clusters and all 4 seed families (Additional file 5: Figure S3). Among the miRNAs that antagonize the miR-17 group in all three cancers, only two were shared. In all 6 analyses (three cancers, positive and negative correlations) miR-221 was present. The highly related miR-222 was found in 5 of the 6 analyses (Additional file 9: Table S8-2). We therefore conclude that in all three cancers miR-221/222 antagonize the miR-17 family.

### The miR-17 group and miR-221/222 are antagonistic in three cancers

**Figure 4:**
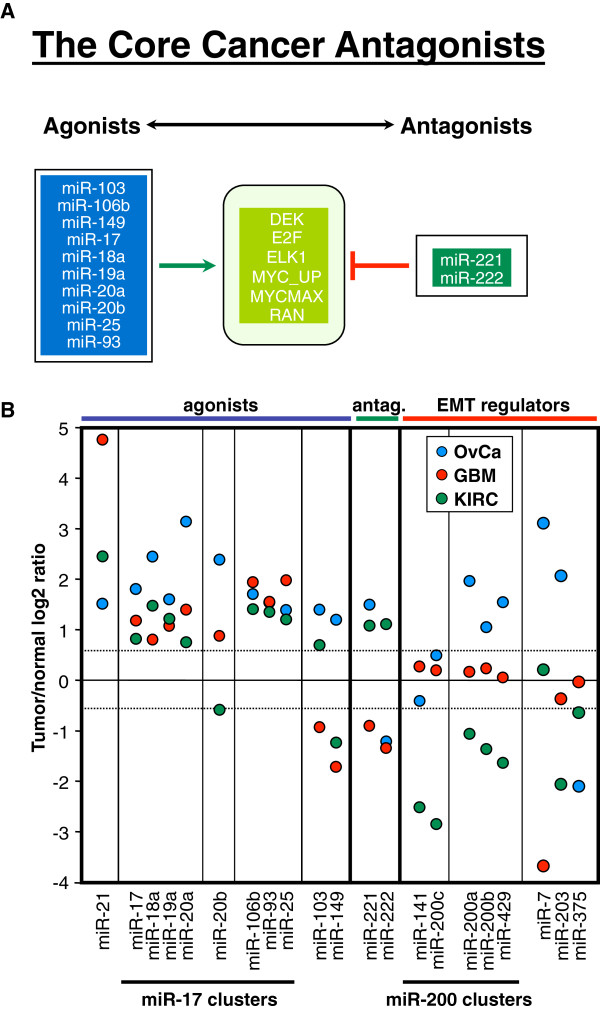
**Agonistic and antagonistic miRNAs and their correlating oncogenic gene signatures in three primary cancers.** (**A**) Correlation network between agonistic miRNAs (blue), antagonistic miRNAs (dark green), and 6 oncogenic signatures (light green) shared by OvCa, GBM, and KIRC. (**B**) Tumor/normal log2 ratio of miRNA expression in primary cancers. From left to right: agonists, antagonists, and EMT regulators. miR-21 is included as a positive control as a miRNA that is upregulated in most human cancers. Stippled lines indicate the 1.5 fold up- or downregulation, taken as the cut-off for genes deregulated in the cancers when compared to matched normal tissues.

**Figure 5:**
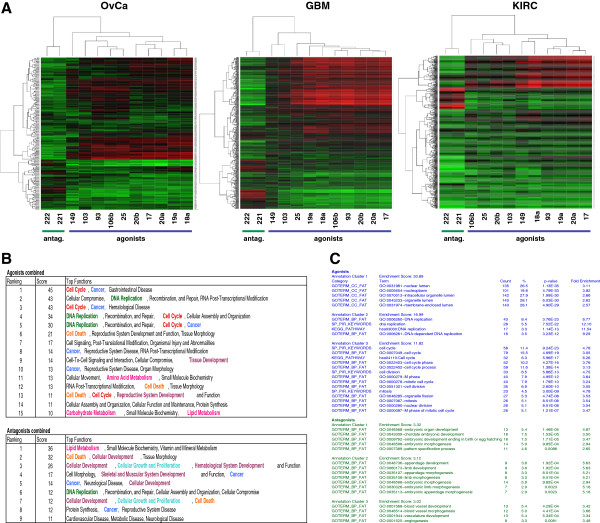
**Functional analysis of genes correlating with agonistic and antagonistic miRNA clusters.** (**A**) Heat maps displaying nonhierarchical unsupervised cluster analyses of the sPCC values derived by comparing the expression of 10 agonistic and 2 antagonistic miRNAs with the combined genes of the 6 oncogenic signatures listed in Figure 4A (data are found in Additional file 13: Table S12). (**B**) IPA network analysis of the combined genes (from the 6 oncogenic gene signatures) that positively correlated with the agonistic miRNAs (upper panel) or antagonistic miRNAs (lower panel). (**C**) DAVID gene ontology analysis of the same genes analyzed in B. Lists of genes used for these analyses can be found in Additional file 11: Table S10.

The agonistic group of miRNAs (containing miR-17 family members) correlated inversely with a number of oncogenic signatures when compared to the antagonistic group (containing miR-221/222). While there were many cancer specific links, we sought to identify correlations that were independent of cancer type. Figure 4A presents the miRNAs and the oncogenic gene signatures that inversely correlated with the agonists and the antagonists in all three cancers. Ten agonistic miRNAs (blue) and two antagonistic miRNAs (dark green) that correlate with genes in six gene signatures (light green) operate in opposition. A number of these oncogenes are known to be connected to the miR-17 family. miR-17 is known to be regulated by and to regulate c-Myc and E2F [32,36-39]. In addition, DEK is regulated by E2F [40]. It is at present unknown how miR-221/222 is linked to either c-Myc or E2F. While miR-17 and miR-221/222 are functionally antagonistic, they are both often upregulated in cancer, and both are considered to be oncogenic in cancer cells [41-65]. Functional antagonism does not necessarily mean that they exhibit inverse expression levels. When compared to the expression in normal control tissue, the expression of miR-221/222 in KIRC was as high as that of miR-17 ~ 92 cluster miRNAs (Figure 4B). These data suggested that the agonistic and antagonistic miRNAs, while both potentially oncogenic, may regulate distinct sets of genes associated with different tumorigenic pathways. In order to test this hypothesis, we combined all genes that were part of the six gene signatures antagonized by the two miRNA groups. For each of the 12 agonistic/antagonistic miRNAs and for each of the three cancers, we calculated the sPCC for all individual miRNA/mRNA correlations. The data were subjected to a two-dimensional unsupervised hierarchical cluster analysis according to the similarities of sPCCs (Additional file 10: Table S9). The results of this analysis are shown in form of heat maps in Figure 5A. In each cancer, all 10 agonistic miRNAs (miR-17 family members, miR-103 and miR-149) were clustered, as were the antagonistic miRNAs (miR-221/222). In general, the genes that positively correlated with the agonists negatively correlated with the antagonists and vice versa. This finding is consistent with the hypothesis that agonistic and antagonist miRNAs contribute to different activities of cancer cells.

### Oncogenic agonists and antagonists regulate distinct cancer specific pathways

**Figure 6:**
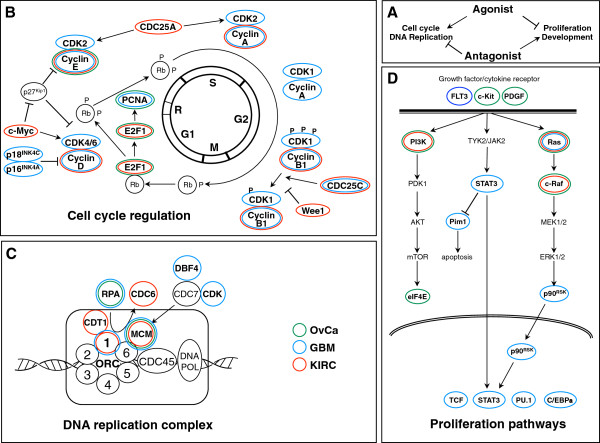
**Cancer relevant signaling pathways that the agonistic and antagonistic miRNAs may regulate in human cancers.** The lists of genes that positively correlate with either the agonistic or the antagonistic miRNAs (Additional file 11: Table S10) were subjected to an IPA analysis. (**A**) Genes that inversely correlated with agonists and antagonists fell into four classes, cell cycle regulation, DNA replication, proliferation, and development. (**B**, **C**) Genes related to cell cycle regulation (**B**) and DNA replication (**C**) whose expression was positively correlated with the agonistic miRNAs and negatively correlated with the antagonistic miRNAs. (**D**) Genes linked to proliferation whose expression was positively correlated with the antagonistic miRNAs and negatively correlated with the agonistic miRNAs. (**B**, **C**, **D**) The three different colors indicate the three different cancers: green, OvCa; blue, GBM; red, KIRC.

To identify the genes that are most highly correlated with either the agonists or the antagonists and to perform pathway analysis, we ranked all signature genes according to the difference (delta) between the average sPCC of the 10 agonists and the average sPCC of the 2 antagonists (Additional file 10: Table S9). To focus on the most significantly correlating genes, we selected all genes that had a delta > 10 or < −10. The lists of agonistic or antagonist genes for each cancer and for all cancers combined are provided in Additional file 11: Table S10. To explore the possible functions of these genes, we subjected each list to pathway analysis. Ingenuity integrated pathway analysis (IPA) revealed that genes whose expression positively correlated with expression of the agonists fell into functional networks that were consistent with cell cycle regulation and DNA replication (Figure 5B). In contrast, genes whose expression positively correlated with expression of the antagonists fell into functional networks that included various forms of developmental processes and cellular growth and metabolism (Figure 5B). This was true for each individual cancer as well as for the combination of the three cancers (Additional file 12: Table S11). Similar results were found when the same genes were subjected to gene ontology analysis using the Database for Annotation, Visualization and Integrated Discovery (DAVID) (Figure 5C). The top three most highly enriched clusters of genes that correlated with the agonist groups of miRNAs (most prominently miR-17) suggested that the agonistic miRNAs positively regulate cell cycle and DNA replication. In contrast, the top three most highly enriched clusters of genes that correlated with the antagonists (miR-221/222) suggested that the antagonistic miRNAs are involved in different developmental processes (Figures 5C and 6A). Using the pathway analysis tool in the Ingenuity IPA, we analyzed the pathways most prominently aligned with the regulated gene sets. The pathways most prominently positively correlated with the agonistic miRNAs were cell cycle regulation and the DNA replication complex (Figure 6B and C). In contrast, some of the most prominent pathways that correlated with the antagonists include growth factor receptor signaling pathways consistent with the growth promoting activity assigned to these miRNAs (Figure 6D). In summary, we have identified two opposing miRNA groups, both of which are oncogenic, presumably by regulating different cancer relevant pathways.

### Different patient populations are dominated by either the agonists or the antagonists

**Figure 7:**
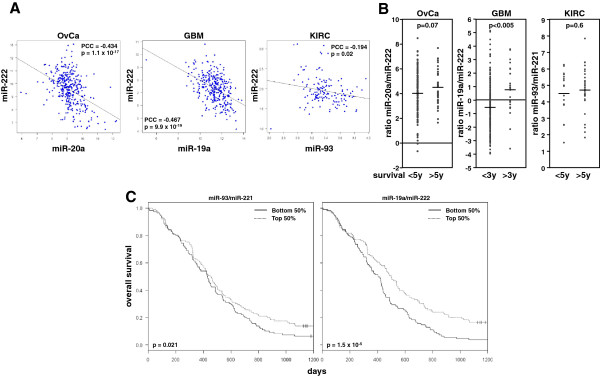
**The most negatively correlated agonist/antagonist miRNA pairs influence patient survival.** (**A**) Correlations and regression analyses between the most negatively correlated agonist/antagonist pairs in primary cancers (all correlations are summarized in Additional file 13: Table S12). Each dot denotes a patient. (**B**) Expression ratios of the most negatively correlated agonist/antagonist pairs in primary cancers grouped according to the patient survival (< 5 years and > 5 years for OvCa and KIRC; < 3 years and > 3 years for GBM). (**C**) Kaplan-Meier survival analysis of GBM patients stratified according to the two most negative agonist/antagonist miRNA pairs (left panel, miR-93/miR-221 ratio; right panel, miR-19a/miR-222 ratio). Solid lines, survival of patients with agonist/antagonist miRNA ratios in the bottom half; dashed lines, survival of patients with agonist/antagonist miRNA ratios in the top half.

How do we explain the paradox that for each of the three cancers two miRNA groups were identified that had opposing activities yet are both considered to be oncogenic, and both are correlated with distinct sets of oncogenic genes? There are three, not necessarily mutually exclusive, possible explanations: 1) The patient population is heterogeneous, and in some patients tumors are driven mostly by miR-17 miRNAs and in others tumors are driven by miR-221/222. 2) Tumors are heterogeneous, and areas within the tumors contain cells that are predominantly regulated by one or the other miRNA group. 3) Both miRNA groups are active in most tumor cells but regulate different sets of tumorigenic genes. To get a preliminary answer, we analyzed the patient data attached to each data set in our analyses. Assuming that the expression of either the agonists or the antagonists would differentiate two patient populations, we performed a one-by-one regression analysis for each of the 10 agonists with either miR-221 or miR-222 in each cancer (Additional file 13: Table S12). Interestingly, all correlations with p <0.05 exhibited a negative PCC suggesting that agonists and antagonists within each cancer type were indeed inversely correlated. The most significantly negatively correlated miRNA pairs for each cancer are shown in Figure 7A. For OvCa this was miR-222/miR-20a; for GBM it was miR-222/miR-19a, and for KIRC it was miR-221/miR-93. The most significant inverse correlation was seen in the GBM pair. To test whether the predominance of either agonists or antagonists was prognostic, we analyzed patient data for each of the miRNA pairs shown in Figure 7A and Additional file 10: Table S9. In each case, a high agonist/antagonist ratio was associated with better overall survival (Figure 7B). For KIRC this was only a trend, and for OvCa it was a strong trend, almost reaching significance, but for GBM it was significant. For GBM we performed a Kaplan-Meier analysis and again found that patients with a high agonist/antagonist ratio had a more favorable outcome. Specifically, we analyzed the effect of the two most significantly inversely correlated miRNA pairs (miR-93/miR-221 and miR-19a/miR-222, see Additional file 13: Table S12-2). Patients with high miR-93/19a expression had a more favorable outcome compared to patients with high miR-221/222 expression (Figure 7C). Our data suggest that while it cannot be excluded that agonists and antagonists act in the same cells or in different areas of the tumor, patients express different ratios of agonists/antagonists, and the predominance of the miR-221/miR-222 oncogenic miRNAs results in poorer outcome than does predominance of miR-17 family members.

## Discussion

In this work we have identified groups of miRNAs that antagonize each other in cancer cells beyond the simple concept of miRNAs as either being tumor suppressors or oncogenes. This became apparent from our analysis of data sets from the NCI60 cells, which are the basis of the miRConnect.org site. A large group of miRNAs containing mostly members of the three miR-17 gene clusters (highlighted in blue in Figure 1A, B, and C) functionally antagonized a group of miRNAs whose expression correlated with mesenchymal genes. This group of mesenchymal miRNAs, in turn, antagonized miRNAs that have been shown to be expressed in epithelial tissues including all members of the miR-200 family (highlighted in red in Figure 1A, B and C). The epithelial miRNAs also included three miRNAs we previously identified as novel epithelial regulators, miR-7, miR-203, and miR-375 (highlighted in orange in Figure 1C).

We then extended this analysis from the NCI60 cells to three primary cancers, OvCa, GBM, and KIRC. We employed the summed PCC analysis we recently developed [4]. Unlike the NCI60 cells data sets, normal tissue of similar tissue origin were available for all three cancers. This allowed us to focus the analysis only on miRNAs and mRNAs that were deregulated (>1.5) in cancer, thereby making the entire analysis cancer specific. We have updated miRConnect.org to also include the data on the three cancers.

In all analyses, most members of the miR-17 family were clustered with miRNAs that correlated with c-Myc regulated genes and genes that are part of E2F gene signatures. This is consistent with the regulation of miR-17 by c-Myc and with the fact that miR-17 is part of a regulatory network with E2F (see discussion below). Because this strong connection was found in the NCI60 cell based analysis and in the three primary cancers, we labeled these miRNAs as agonists. In all analyses the agonistic miRNAs were separable from and opposite to a group of miRNAs that negatively correlated with c-Myc induced genes. In contrast to the agonists, no single miRNA was shared by all antagonistic miRNA groups in all analyses. However, two miRNAs, miR-221 and miR-222, were found in this antagonistic group in all three primary cancers pointing at differences between cell lines and primary cancers. Such differences were also evident in comparisons of correlations involving the epithelial miRNAs. Although all 5 miR-200 family members, coded by two different gene clusters, were tightly clustered in the NCI60 cell lines, the situation was much different in the three primary cancers (Figure 2). In OvCa, only miR-375 was part of the cluster with the strongest epithelial nature. In GBM, none of the miR-200 family members or novel EMT regulators were found to be deregulated. However, in KIRC all 5 miR-200 family members were found to be part of a highly epithelial miRNA cluster, but within this large group they clustered according to their chromosomal localization. There could be a variety of reasons why the miR-200 family clustered so tightly in the NCI60 analysis. For example, it might be a consequence of the fact that the cells have been cultured on plastic for a long time. It could also be that the NCI60 cell lines represent a more homogeneous group of cells considering that they are all cancer cell lines. However, among the cell lines there is tremendous variation with respect to their epithelial nature. The analysis may simply highlight these biological differences suggesting that the NCI60 analysis may be more useful for isolating general biological connections rather than cancer specific properties.

The agonist miRNAs that we found to correlate with a large number of oncogenic gene signatures were dominated by members of the three miR-17 gene clusters (Additional file 5: Figure S3). In contrast, miR-221 and miR-222 were found in the antagonistic miRNA group in all three cancers. Paradoxically, both miRNAs families are considered to be oncogenic. The miR-17 ~ 92 cluster of miRNAs was originally identified as being amplified in B cell lymphoma patients. Consequently, most of the data on miR-17 miRNAs are in the context of its role as an oncogene in blood cancers. Early on, a correlation with c-Myc expression was noticed, and enforced expression of miR-17 ~ 92 accelerated B cell lymphoma formation in mice [66]. Subsequently, it was recognized that c-Myc activates the miR-17 ~ 92 cluster [32,36,37]. miR-17 was identified as part of an autoregulatory loop with E2F proteins. While all three E2Fs can activate the miR-17 ~ 92 promoter [38], E2F2 and E2F3 are also targets of miR-17 ~ 92 miRNAs [39]. Overexpression of miR-17 ~ 92 has also been reported in solid cancers including lung cancer [41,42], colon cancer [43,44], thyroid cancer [45], gastric cancer [46], nasopharyngeal carcinoma [47], hepatocellular carcinoma [48], lung squamous cell carcinoma [49], malignant glioma [50], and pancreatic cancer [51]. In fact, miR-17 ~ 92 was reported to be a component of a solid cancer miRNA signature [52]. Of the two non-miR-17 family members (miR-103 and miR-149) among the agonists in our study, miR-103 is upregulated in bladder cancer [59], esophageal squamous cell carcinoma [67], gastric cancer [68], and colon cancer [69]. Little is known about the role of miR-149 in cancer. Similar to miR-17, miR-221 and/or miR-222 are highly upregulated, often without concurrent upregulation of miR-17 ~ 92, in various cancers including glioblastoma [53], liver cancer [54], pancreatic cancer [55-58], bladder cancer [59], gastric cancer [60,61], ovarian cancer [62], urothelial carcinoma [63], nodal marginal zone lymphoma [64], and papillary thyroid carcinoma [65].

Our pathway analyses suggest that while both agonists and antagonists are oncogenic, they regulate different oncogenic signaling pathways, each of which may contribute to cancer development or metastases by different mechanisms. Different mechanisms of action for the agonists and antagonists are supported by a substantial body of published work. c-Myc driven miR-17-92 expression has been shown to promote tumor angiogenesis [36], and inhibition of miR-17-5p and miR-20a induces apoptosis in lung cancer cells [70] and leads to induction of apoptosis, cellular senescence, and growth inhibition of thyroid cancer cells [45]. miR-17 ~ 92 drives proliferation by targeting a number of cell cycle regulators of the G/S transition [71]. Deletion of the miR-17-92 cluster in mice resulted in increased levels of the apoptosis inducer Bim. The mice died after birth, and exhibited lung hypoplasia and lack of B cell development [72]. In humans, a germline deletion of miR-17-92 causes skeletal and growth defects [73]. miR-17 has been implicated in tumor angiogenesis, cell cycle, and cell death regulation, while miR-221/222 has been linked to cell proliferation in cancer. Inhibition of endogenous miR-221/222 impaired growth of prostate carcinoma xenografts in mice [74], inhibited the growth of liver cancer [54], and arrested pancreatic cancer cells in cell cycle driving them into apoptosis [75]. Anti-miR-221 treatment in an orthotopic HCC mouse model blocked cancer by reducing proliferation [76]. In contrast, overexpression of miR-221 in a mouse model of liver cancer stimulated growth of tumorigenic murine hepatic progenitor cells [54], and miR-221/222 increased proliferation of ER positive breast cancer cells [77], gastric cancer cells [78], and GBM [79]. Most significantly, transgenic overexpression of miR-221 alone caused HCC, and anti-miR-221 treatment reduced tumor load [80]. miR-221/222 has been shown to affect cancer proliferation by targeting p27 Kip1 [81]. This was specifically shown for prostate cancer [82], melanoma [83], HCC [84], and breast cancer [85,86]. In addition, activation of the Akt pathway has also been reported [87,88]. Our analysis suggests that miR-221/222 does not regulate proliferation, but plays a role in development. A connection between miR-221 and development has been reported; overexpression of anti miR-221 in human embryonic stem cells and mesenchymal stem cells triggered osteogenic differentiation [89].

Our analysis indicates that the distinction between miRNAs as oncogenic or tumor suppressive does not adequately describe their functions. In fact in this report, we have identified two miRNA groups that are oncogenic and, yet, are functional antagonists across three different human cancers. Given the fact that the two groups of miRNAs antagonized a large portion of the genes that comprise 158 oncogenic signatures included in the analysis, it is possible that the two miRNA groups act in the same cells. However, by comparing different patients, we found that in some patients, expression of miR-17 family members predominated, whereas in others miR-221/222 predominated. This was especially obvious for GBM. In all three cancers, patients with a high miR-221/222 to miR-17 ratio had poorer long term survival. In GBM the difference was significant. In GBM, a high miR-93/miR-221 or a high miR-19a/miR-222 ratio was predictive of better overall survival. There was no correlation between the miR-17 to 221/222 ratio and tumor grade or stage (data not shown) suggesting that the ratio of agonists to antagonists does not change much during tumor progression, but does suggest that different miRNAs are expressed in different patients.

## Conclusions

Numerous studies have assigned cancer relevant activities to miRNAs using both miRNA and mRNA profiles in either NCI60 cell lines or in primary tumors (i.e., derived from TCGA [15,16]). Most of these studies have used Pearson’s Correlation Coefficients and/or target prediction algorithms to identify targets of individual miRNAs in a specific cancer background. A few studies have identified common or specific miRNA functions across tumors of multiple origins by applying various statistical models [25-28] or by analyzing oncogenic signatures [29]. The primary aim of most of these studies was to predict and validate novel cancer relevant miRNA targets. In contrast, our work across the NCI60 cell lines and three primary cancers has focused on miRNA downstream effector genes without considering target prediction, and hence, effectively avoids the highly false positive rate produced by target prediction algorithms. Our method permits extraction of statistically solid and biologically relevant miRNA-mRNA pairs on a genome-wide scale. In so doing, we have identified functionally defined miRNA groups which have opposing activities in cancers, yet can both be considered to be oncogenic. These activities are not specific for individual cancers, and suggest that they reflect fundamental activities of miRNAs in human cancers.

## Methods

### The Cancer Genome Atlas (TCGA) data sets

The TCGA database (https://tcga-data.nci.nih.gov/tcga/) was used to extract gene and miRNA expression data sets from different solid cancers. There were a total of 19 available solid cancer types in the TCGA database. In order to compare data derived from high quality tumor material, the following high stringency selection criteria were applied: 1) Only cancers with data sets of more than 100 patients were considered. 2) Only patients for whom complete mRNA and miRNA data sets were available were included. 3) Only patients for whom a pathologist had determined the percent tumor cells by histological evaluation of one section taken from the top and one from the bottom of the tissue block were considered. Only tumor samples with >70% tumor cells (as an average between top and bottom analyses) were included. 4) Additional cancer specific criteria were applied to focus on the most relevant and most homogeneous groups of tumor tissue for each cancer (for details see Additional file 4: Table S4, column 6). Four cancers met these criteria, breast invasive cancer (BrCa), Glioblastoma multiforme (GBM), kidney renal clear cell carcinoma (KIRC), and ovarian serous cystadenocarcinoma (OvCa). Eventually, BrCa was excluded from the analysis because its high degree of heterogeneity did not permit meaningful analysis using the sPCC method (data not shown). Gene and miRNA expression data sets of matching normal samples from GBM, KIRC, and OvCa were also extracted from the TCGA database. Normal tissues are matched to the anatomic site of the tumor but usually not matched to the participant. The number of normal tissues for each cancer is given in Additional file 4: Table S4, column 7.

### Selection of deregulated miRNAs and mRNAs in primary cancers

For each cancer, the tumor/normal ratio of each miRNA or gene was calculated using the average expression value in the respective samples. Both fold cutoff (≥ 1.5, up or down) and p-value cutoff (two-sided T-test, p < 0.05) were employed to identify the significantly deregulated miRNAs and mRNAs in the three cancers. This procedure removed about half of the miRNAs and mRNAs to be analyzed thereby reducing noise. Details on deregulated miRNAs and mRNAs are found in Additional file 14: Table S13.

### Statistical and data analyses

Unless otherwise stated, all statistical analyses of data including gene expression data manipulation, sPCC calculations, hierarchical clustering, PCA analysis, and gene expression signature calculations were performed using R statistical program v2.10 (http://www.r-project.org/).

### Method to identify correlations between miRNAs and mRNAs in primary cancers

To identify significant correlations between miRNAs and mRNAs we employed a modified form of the Pearson's Correlation Coefficient, called summed (s)PCC, that we recently described [4]. In short, for each cancer type, from the TCGA expression data we selected “deregulated” miRNAs and mRNAs whose expression differed by at least 1.5 fold when compared to normal tissue. Using these deregulated miRNAs and mRNAs, patients were ranked according to their miRNA expression levels from highest to lowest. This ranked list of patients was used to generate deregulated subsets of patient expression data, which we call “patterns”. For each miRNA, the patternX/2 (X being the total number of miRNA data sets for each cancer) consisted of the top half of patients (those with the highest levels of miRNA expression), patternX/2 + 1 included all of the patients from patternX/2 and the patient with the next highest level of expression, patternX/2 + 2 included all of the patients from patternX/2 + 1 and the patient with the next highest level of expression, and so on. The last pattern, patternX, consisted of all of the patients, and completed the set of deregulating patterns. Each individual pattern was used as a seed for a single PCC calculation between each miRNA and mRNA. For each miRNA, PCCs of all patterns were added up resulting in the sPCC value. To generate hierarchical clustering of miRNAs for each cancer, the top 2000 genes (covering about 10% of all genes) with the most highly positive or negative sPCCs were used. For all analyses involving the primary cancers, different sPCC cut-offs were chosen (±5 for OvCa, ±6 for GBM, and ±2 for KIRC) to permit comparison of the data with those generated for the NCI60 cells for which we had used a cut-off of ±1 [4]. These cut-offs were proportional to the different samples sizes of each data set (NCI60 = 59; OvCa = 320; GBM = 353; KIRC = 142).

### Principal component analysis of miRNAs

The Principal Component Analysis (PCA) correlating miRNA expression with mRNA expression in the NCI60 cells was published recently [4]. In short, expression data (the 2000 most positively correlating genes) for each of the 136 miRNAs significantly expressed in NCI60 cells were used to perform a PCA analysis. A 136 × 136 matrix of overlapping gene numbers between miRNAs (recently described [4]) was used to calculate principal components (PCs). Of the 136 PCs, the first two combined covered about 50% of all variance between miRNAs (data not shown).

### Gene signatures

The three different EMT signatures as well as Myc-induced and Myc-repressed signatures used in this work were reported in our previous analysis [4]. As a modification, we now generated an average EMT signature by combining the three previously described EMT signatures. For each miRNA, the value of the normalized EMT signature was calculated as the average value of the 3 EMT signatures. We also combined the two previously described individual signatures of Myc-induced and Myc-repressed genes [4] into one Myc signature. To generate this normalized Myc signature for each miRNA, a value was calculated as: (positively correlated Myc-induced genes – neg. correlated Myc-induced genes) / total # of Myc-induced genes - (positively correlated Myc-repressed genes – neg. correlated Myc-repressed genes) / total # of Myc-repressed genes. A total of 158 oncogenic signatures (see Additional file 1: Table S1) were used to identify connections between miRNAs and oncogenesis. These signature lists were curated from several sources including the Broad Institute, Biocarta, Johns Hopkins University, and selected studies derived from PubMed (see Additional file 1: Table S1 for details and PubMed IDs). We determined whether expression of genes in all these lists negatively or positively correlated with the expression of deregulated miRNAs in each primary cancer. For each signature, the number of genes whose expression negatively correlated with a given miRNA was subtracted from the number that positively correlated, and the result was plotted across all deregulated miRNAs in the sPCC-based cluster analysis. Statistically significant correlations between gene expression and the expression of miRNAs in each functional cluster were determined using the Wilcoxon Rank-Sum Test (p < 0.01).

### Overlap matrix

Overlap matrixes were generated to identify miRNA groups that are functionally antagonistic to each other. A total of 16 such overlap matrices were produced (Additional file 2: Tables S2, Additional file 3: Table S3, Additional file 7: Table S6 and Additional file 8: Table S7). To generate an overlap matrix, first the significantly positive or negative correlations between each miRNA cluster and EMT or oncogenic signatures were calculated (see bottom table in Additional file 2: Tables S2, Additional file 3: Table S3, Additional file 7: Table S6 and Additional file 8: Table S7). In these tables, rows and columns correspond to the miRNA functional clusters. The upper right half of each table contains the agonistic correlations (which were not further considered), and the lower left half contains the antagonistic correlations. The diagonal was defined as Not Available (NA). The first number in each cell represents the number of gene signatures for which an antagonistic correlation was found (p < 0.001). A single antagonistic correlation was defined as one miRNA cluster that positively correlated with a gene signature and another cluster that negatively correlated with the same gene signature. The second number in each cell shows the number of gene signatures for which a significant correlation (p < 0.001) (either negative or positive) with a miRNA cluster was found. The third number in each cell (in brackets) represents number 1/number 2 x 100 (=%). miRNA clusters were scored as antagonistic when the numbers and percentages were above a threshold (as defined in the tables) for both overlap matrices generated using positive and negative sPCCs. In the analysis of oncogenic signatures, miRNA clusters were considered only if they were found to correlate with at least 30 different oncogenic signatures (including both positively and negatively correlating genes) and if there was an antagonizing group of miRNAs that fulfilled the same criteria. In the EMT analysis, clusters were considered only if they correlated with at least 2 of the three EMT signatures (including both positively and negatively correlating genes) and if there was an antagonizing group of miRNAs that fulfilled the same criteria. Further details are found in the Additional file tables. miRNA clusters that were identified as being antagonistic are highlighted in different colors (columns at the top of each Additional file table). For the analyses involving oncogenic signatures (Additional file 2: Table S2-2, Additional file 3: Table S3-2 and Additional file 8: Table S7), the actual gene signature lists that were found to be antagonized by miRNA groups are shown at the top right of each table. Each column of signatures refers to a spread sheet cell in the overlap matrix table on the bottom left. Gene signatures that positively correlate with agonistic miRNAs (containing miR-17 family members) are highlighted in green, and signatures that negatively correlate are highlighted in red. The workflow of the entire analysis is illustrated schematically in Additional file 5: Figure S4.

### Ingenuity integrated pathway and DAVID gene ontology analyses

To analyze sets of genes with respect to their possible role in diverse biological signaling pathways, lists of genes that negatively and positively correlate with miRNAs (see Additional file 11: Table S10) were subjected to an analysis using Ingenuity’s IPA application (version 1.0; Ingenuity.com). Both Network analysis and Pathway analysis were performed. The same lists of genes were also analyzed using the DAVID Bioinformatics Resources 6.7 (http://david.abcc.ncifcrf.gov). Gene lists were uploaded to DAVID and subjected to a functional annotation analysis using default settings.

### Survival analysis

Kaplan-Meyer survival analysis was performed to test the influence of agonistic and antagonistic miRNAs on patient survival. For each primary cancer, the most significantly negatively correlated agonistic/antagonistic miRNA pairs were selected, and ratio values across all patients were divided into two groups: higher (top 50%) and lower (bottom 50%) agonistic/antagonistic ratio. The survival time after treatment for each patient was extracted from available clinical information (Additional file 15: Table S14). Patients with incomplete clinical data (e.g., living patients treated within last 3 years, or patients without follow-up information) were excluded. A parametric model with Weibull hazard distribution [90] was constructed to test the difference between two groups for 3-year survival (p < 0.05).

## Competing interests

The authors report no competing interests.

## Authors’ contributions

YH performed the data analysis. NL and JK established and updated the miRConnect site. SKS and AMC selected and provided the oncogenic gene signatures and MEP conceived and designed the experiments, analyzed data and wrote the manuscript. All authors read and approved the final manuscript.

## Supplementary Material

Additional file 1: Table S1 Oncogenic signatures used in the study. Click here for file

Additional file 2: Table S2 Correlation of positively correlating miRNA clusters of the NCI60 Q data with 3 EMT signatures. Click here for file

Additional file 3: Table S3 Correlation of negatively correlating miRNA clusters of the NCI60 Q data with 3 EMT signatures. Click here for file

Additional file 4: Table S4 The TCGA data sets used in the study. Click here for file

Additional file 5: Figure S1 Venn Diagrams of overlapping miRNAs or mRNAs deregulated >1.5 fold in OvCa, GBM and KIRC. **Figure S2.** Scheme illustrating the flow of the analysis. **Figure S3.** The genomic clusters and seed families of the miR-17, miR-221/222 and miR-200 families. **Figure S4.** Functional clusters of deregulated cancer relevant miRNAs in three primary cancers according to positively correlated genes based on the sPCC method. Click here for file

Additional file 6: Table S5 miRNA clusters in all data sets based on positively and negatively correlating genes. Click here for file

Additional file 7: Table S6 Correlation of positively correlating miRNA clusters of the OvCa data with 3 EMT signatures. Click here for file

Additional file 8: Table S7 Correlation of positively correlating miRNA clusters of the OvCa data with 158 oncogenic signatures. Click here for file

Additional file 9: Table S8 miRNAs that are found in the the agonistic miRNA groups in different cancers. Click here for file

Additional file 10: Table S9 sPCC analysis of agonistic and antagonistic miRNAs as shown in Figure 5A-OvCa. Click here for file

Additional file 11: Table S10 Lists of genes that positively correlale with the agonists and antagonists in three cancers. Click here for file

Additional file 12: Table S11-1 Functional networks covered by genes correlating with the agonists. **Table S11**-**2.** Functional networks covered by genes correlating with the antagonists. Click here for file

Additional file 13: Table S12 Correlations between individual agonistic and antagonistic miRNAs in OvCa. Click here for file

Additional file 14: Table S13 Overlap of deregulated miRNAs and mRNAs in the three cancers. Click here for file

Additional file 15: Table S14 Analysis of patient data. Click here for file
