# Supplementary material for: miRConnect 2.0: identification of oncogenic, antagonistic miRNA families in three human cancers
Source: BMC Genomics. 2013 Mar 15;14:179. doi: 10.1186/1471-2164-14-179 (PMC3637148; doi:10.1186/1471-2164-14-179)
Supplement: Additional file 5: Figure S1 — Venn Diagrams of overlapping miRNAs or mRNAs deregulated >1.5 fold in OvCa, GBM and KIRC. Figure S2. Scheme illustrating the flow of the analysis. Figure S3. The genomic clusters and seed families of the miR-17, miR-221/222 and miR-200 families. Figure S4. Functional clusters of deregulated cancer relevant miRNAs in three primary cancers according to positively correlated genes based on the sPCC method. [file 1471-2164-14-179-S5.pdf]

Supplementary Material for

miRConnect 2.0: Identification of oncogenic, antagonistic miRNA families in three human cancers

Youjia Hua[1], Niels Larsen[2], Shanker Kalyana-Sundaram[3], Jørgen Kjems[2], Arul M. Chinnaiyan[3] and Marcus E. Peter[1*]

[1]Feinberg School of Medicine, Division Hematology/Oncology, Northwestern University, Chicago, 60611, USA
[2]Department of Molecular Biology, Aarhus University, Århus, Denmark
[3]Michigan Center for Translational Pathology, Ann Arbor, MI 48109, USA

1. Supplementary Figure 1 - Venn Diagrams of overlapping miRNAs or mRNAs deregulated >1.5 fold in OvCa, GBM and KIRC.

2. Supplementary Figure 2 - Scheme illustrating the flow of the analysis.

3. Supplementary Figure 3 - The genomic clusters and seed families of the miR-17, miR-221/222 and miR-200 families.

4. Supplementary Figure 4 - Functional clusters of deregulated cancer relevant miRNAs in three primary cancers according to positively correlated genes based on the sPCC method.

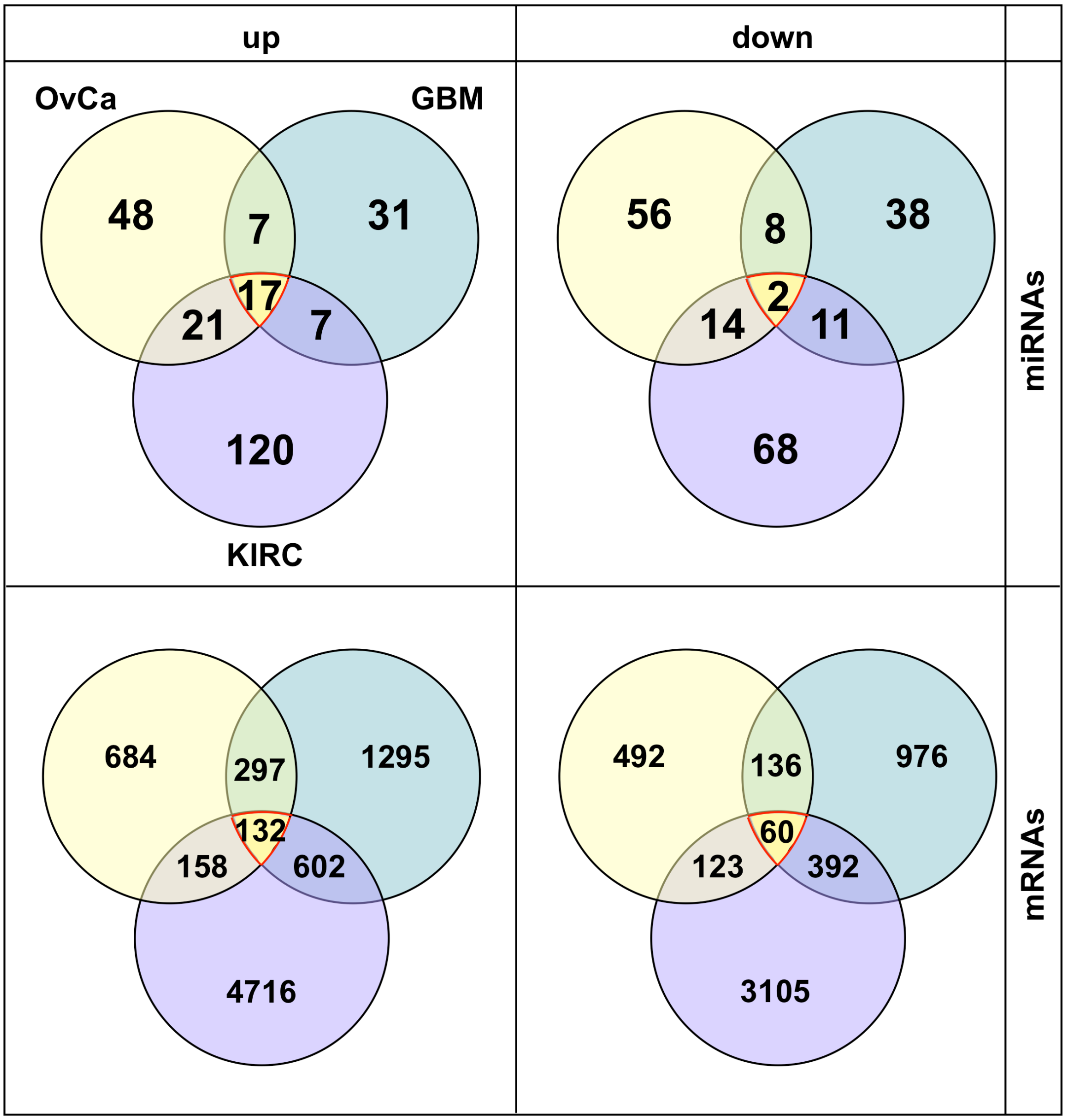

Figure S1 - Venn Diagrams of overlapping miRNAs or mRNAs deregulated >1.5 fold in OvCa, GBM and KIRC. Left two panels, upregulated miRNAs/mRNAs; Right two panels, downregulated miRNAs/mRNAs; Top two panels, deregulated miRNAs; Bottom two panels, deregulated mRNAs. For a break-down of miRNAs and mRNAs for each cancer see Additional file 14: **Table S13**.

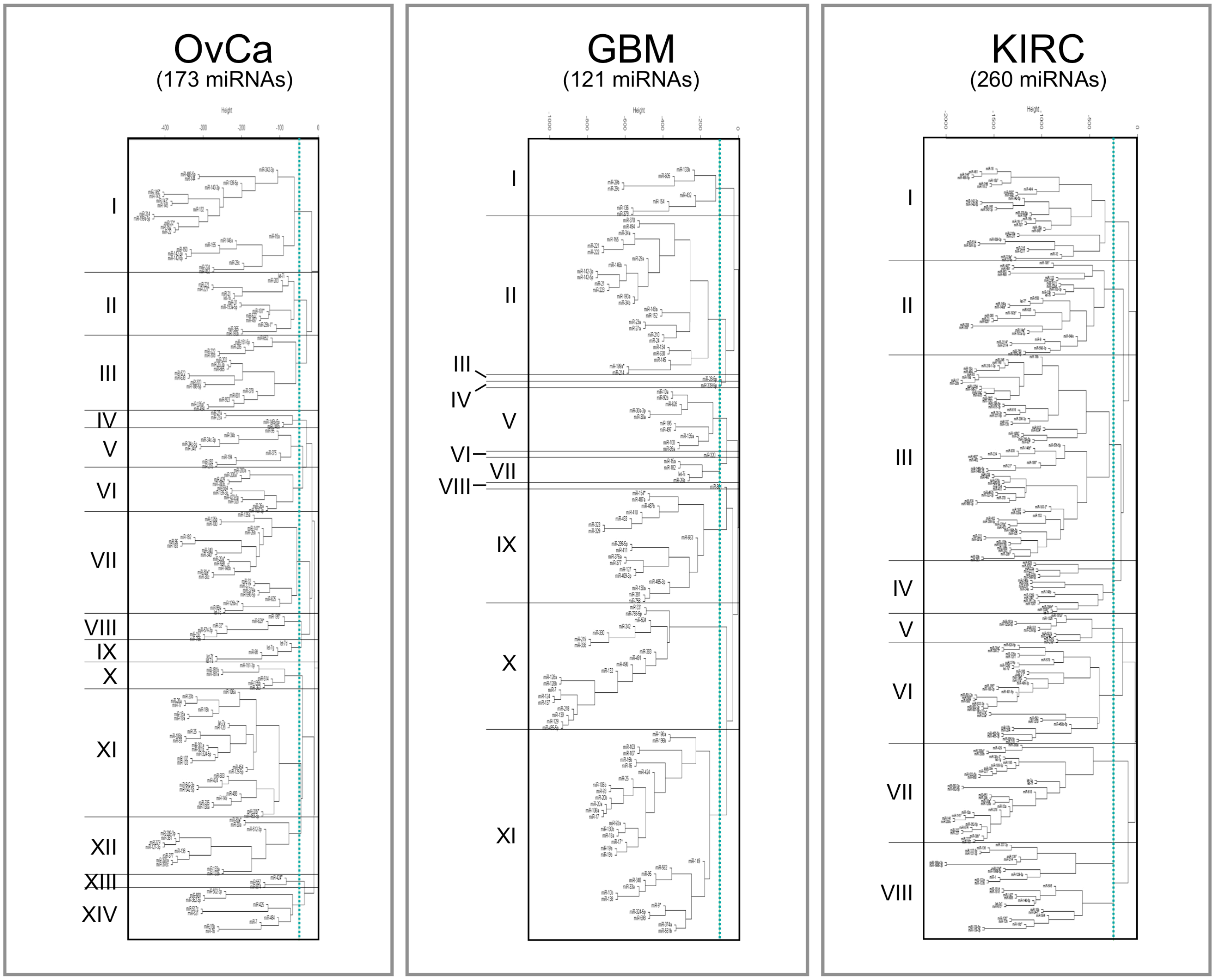

Figure S2 - Scheme illustrating the flow of the analysis. M1-3, the mesenchymal gene signatures and E1-3, the epithelial gene signatures recently described [1].

	Genomic cluster	Seed family
miR-17	miR-17~92 clusters	miR-17~93 family
	Chr #13: miR-17/18a/19a/20a/19b-1/92a-1	miR-17-5p/20ab/93/106ab/427/518-3p/519
	Chr X: miR-106a/18b/20b/19b-2/92a-2/363	miR-18ab
	Chr #7: miR-106b/93/25	miR-19ab
		miR-25,32,92abc,363, 367
miR-221/222	Chr X: miR-222/221	miR-221/222/222ab/1928
miR-200	Chr #1: miR-200b, miR-200a, miR-429	miR-200a, miR-141
	Chr #12: miR-200c, miR-141	miR-200b, miR-200c, miR-429

Figure S3 - The genomic clusters (left column) and seed families (right column) of the miR-17, miR-221/222 and miR-200 families (from top to bottom). Blue, agonistic miRNAs; green, antagonistic miRNAs shared between the three cancers.

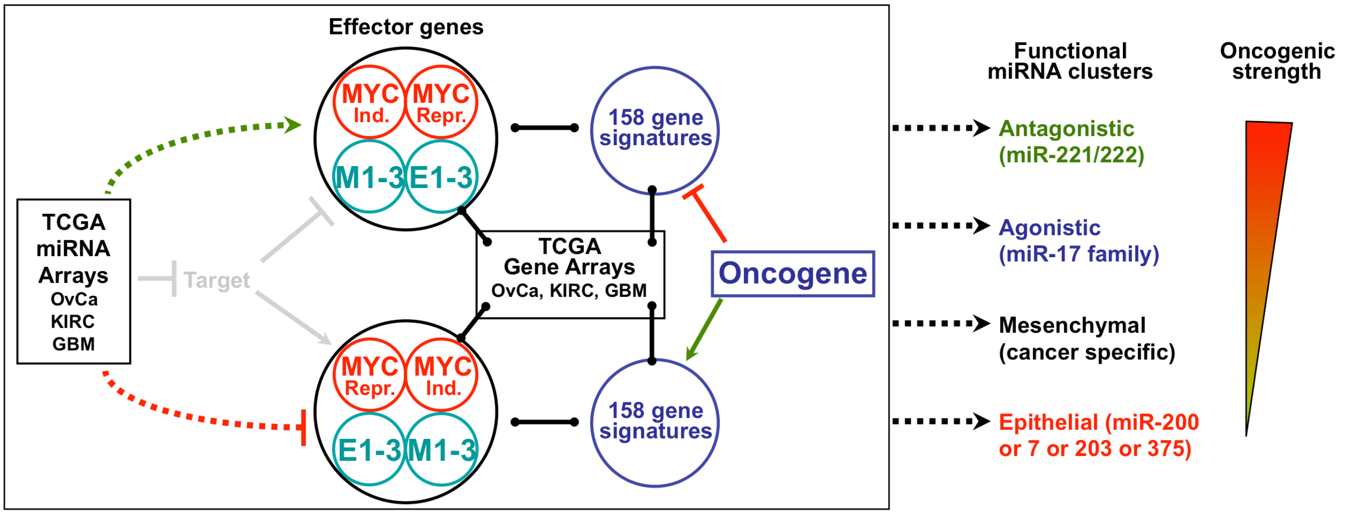

Figure S4 - Functional clusters of deregulated cancer relevant miRNAs in three primary cancers according to positively correlated genes based on the sPCC method. The green stippled line indicates the 12.5% cut-off of overlapping correlating genes that was used to define the clusters.

Supplementary References

1. Hua Y, Duan S, Murmann AE, Larsen N, Kjems J, Lund AH, Peter ME: miRConnect: Identifying Effector Genes of miRNAs and miRNA Families in Cancer Cells. *PLoS ONE* 2011, 6:e26521.
